# Machine Learning to Improve Energy Expenditure Estimation in Children With Disabilities: A Pilot Study in Duchenne Muscular Dystrophy

**DOI:** 10.2196/rehab.4340

**Published:** 2016-07-19

**Authors:** Amit Pande, Prasant Mohapatra, Alina Nicorici, Jay J Han

**Affiliations:** ^1^University of California DavisDepartment of Computer ScienceDavis, CAUnited States; ^2^University of California Davis Health SystemDepartment of Physical Medicine and RehabilitationSacramento, CAUnited States

**Keywords:** accelerometry, physical activity, heart rate, neuromuscular disease, children

## Abstract

**Background:**

Children with physical impairments are at a greater risk for obesity and decreased physical activity. A better understanding of physical activity pattern and energy expenditure (EE) would lead to a more targeted approach to intervention.

**Objective:**

This study focuses on studying the use of machine-learning algorithms for EE estimation in children with disabilities. A pilot study was conducted on children with Duchenne muscular dystrophy (DMD) to identify important factors for determining EE and develop a novel algorithm to accurately estimate EE from wearable sensor-collected data.

**Methods:**

There were 7 boys with DMD, 6 healthy control boys, and 22 control adults recruited. Data were collected using smartphone accelerometer and chest-worn heart rate sensors. The gold standard EE values were obtained from the COSMED K4b2 portable cardiopulmonary metabolic unit worn by boys (aged 6-10 years) with DMD and controls. Data from this sensor setup were collected simultaneously during a series of concurrent activities. Linear regression and nonlinear machine-learning–based approaches were used to analyze the relationship between accelerometer and heart rate readings and COSMED values.

**Results:**

Existing calorimetry equations using linear regression and nonlinear machine-learning–based models, developed for healthy adults and young children, give low correlation to actual EE values in children with disabilities (14%-40%). The proposed model for boys with DMD uses ensemble machine learning techniques and gives a 91% correlation with actual measured EE values (root mean square error of 0.017).

**Conclusions:**

Our results confirm that the methods developed to determine EE using accelerometer and heart rate sensor values in normal adults are not appropriate for children with disabilities and should not be used. A much more accurate model is obtained using machine-learning–based nonlinear regression specifically developed for this target population.

## Introduction

Accelerometry-based algorithms quantifying the energy estimation (EE) or calories-out of users and measuring physical activity of healthy populations are becoming popular in the consumer electronics market [1,2,3]. Smartphone apps and devices such as Fitbit, Jawbone Up, Nike+ Fuelband, Microsoft Band, and Apple Watch use underlying accelerometer sensors and machine-learning algorithms developed on a pool of healthy adults to give real-time EE estimates. Many of these algorithms rely on fusing heart rate measurements with accelerometer readings. It is tempting to use similar algorithms to quantify the EE of children with disabilities. However, to the best of our knowledge, there has been limited effort to validate application of machine-learning–based EE algorithms for pediatric patients with muscular dystrophy. A better understanding of real-world community-level physical activity patterns and EE would lead to more targeted interventions to combat obesity and decreased physical activity in this population.

Different measuring techniques have been used in disabled populations including questionnaires, activity diaries, heart rate monitoring, motion sensors (eg, pedometers, accelerometers), indirect calorimetry, and doubly labeled water. Activity questionnaires and diaries, while inexpensive, are time consuming, rely on recall and reporting by the individual, and have been shown to be inaccurate, especially in children [4,5]. Indirect and direct calorimetry cannot be used in home and outdoor scenarios and are restricted to clinical settings. In healthy normal populations, heart rate monitoring has been shown to be less accurate in estimating EE for low-intensity activities, which comprise the majority of the activity for disabled populations [4,5]. Accelerometers are more accurate for nondisabled populations because they measure activities across several planes allowing measurements of the duration, frequency, and intensity of physical activity. Disadvantages include the inability to measure activities where the patient is not moving the part of the body being monitored by the accelerometer (eg, cycling, sitting, standing) [6]. Development of EE algorithms utilizing inertial sensor (accelerometer) data has thus far been largely restricted to healthy adult populations. Sensor-based EE estimation relies on previously developed general formulas, and no data exists for specific pediatric populations including children with disabilities. Simply extending basic EE estimation algorithms developed for healthy adults for use with children with physical disabilities is problematic.

In this study, we will identify important factors for EE calculation and develop algorithms that accurately estimate EE for a specific target pediatric population, children with Duchenne muscular dystrophy (DMD). These data can then be used to measure community habitual physical activity and EE using sensors.

DMD is one of the most common hereditary (X-linked recessive) neuromuscular disorders affecting the pediatric population and also represents a prototypical muscle disorder with proximal limb girdle weakness that results in a wide spectrum of physical impairments. Its prevalence is approximately 1 per 3500 to 5000 boys, making it the most common and severe form of childhood muscular dystrophy. Boys with DMD are usually confined to a wheelchair by 10 years of age and have a median life expectancy of 30 years [7]. Muscle weakness, followed by muscle and tendon retractions and joint deformities, causes gait impairment in patients with DMD, leading to compensatory movements and gait deformation. The compensatory movements occur because of the selection of possible synergic movements on hip, knees, and ankles and the development of new motor strategies used to allow the maintenance of ambulation [8].

The aim of this work is to test the efficiency of existing regression models (originally built based on data from healthy population samples) on children with disabilities. Since boys with muscular disability (and DMD in particular) perform compensatory movements to walk and have a different body mass composition, it is possible that this population requires a specific model rather than reusing normal models. Existing works have targeted studying resting energy expenditure (REE) in DMD patients and report it to be significantly lower than controls of similar population [9]. Elliott et al [10] predicted REE using existing equations based on anthropomorphic features and fat-free mass. Souza et al [11] estimated EE during ambulatory activities for a study of 3 patients using a linear formula based on heart rate.

## Methods

### Subjects

There were 7 subjects with DMD aged 6 to 10 years recruited from the regional neuromuscular clinic at the UC Davis Medical Center, and 6 control children and 23 healthy adults were recruited locally. Subjects completed an informed written consent approved by the Institutional Review Board of the University of California Davis.

### Experimental Design

Subjects were asked to perform a series of activities in our exercise laboratory at UC Davis while being monitored by an accelerometer, a heart rate monitor, and the COSMED K4b2 (COSMED USA) metabolic system. For accelerometer measurements, we used smartphone devices placed in a waist pack and oriented in a standardized position. A chest strap was used for the heart rate monitor.

### Exercise Protocol

Before each test, the COSMED K4b2 components were calibrated according to the manufacturer’s instructions. Subjects were then fitted with the pack containing the phone (accelerometer) and the COSMED K4b2 metabolic system. Subjects were asked to perform the following activities, one right after the other, in the ordered listed, with approximately 1 minute rest between the walking protocols:

- 3 minutes of lying supine on an exam table
- 3 minutes of sitting
- 50-meter slow-paced walk (lasting approximately 1-2 minutes)
- 50-meter typical comfortable speed walk (45-60 sec)
- 50-meter fast walk (20-60 seconds)

Speeds were chosen based on ratings from the the OMNI scale of perceived exertion with easy walking rated as 0 to 2 or “not tired at all,” medium pace as 2 to 4 or “getting a little tired,” and fast walking pace as 4 to 6 or “getting more tired.” The final activity was a 6-minute walking test. Cones were set up 25 meters apart in the hallway and the children walked as fast as possible back and forth between the cones for 6 minutes. Heart rate (using a Polar heart rate monitor), oxygen consumption, carbon dioxide production, respiratory exchange ratio (RER), and ventilation rate were continuously monitored.

Data from the COSMED metabolic system were averaged over the 30 to 60 seconds of each collection period. Energy expenditure was calculated using the following equation: COSMED K4b2 EE (kcal/min)=([1.2285*RER]+3.821)*VO_2_ where VO_2_ is the oxygen consumption in liters per minute. All data were processed according to the following procedures:

1. COSMED output was resampled to obtain per-second estimates of EE and heart rate.

2. Smartphone sensors were oversampled at 4 Hz and then downsampled to obtain higher frequency resolution (more accurate sensor readings). Oversampling improves resolution and reduces noise in the readings. Resampling was done to obtain per-second estimates of accelerometer readings (Ax, Ay, and Az relative to the x, y, and z axis of the smartphone).

3. Accelerometer readings were synced with the COSMED readings using paper markers.

Local coordinates from the smartphone accelerometer readings were translated into global coordinates (two components: horizontal and vertical).

4. Additional information about subject measurements such as age, height, and weight were used as attributes for training data-mining algorithms and validating existing algorithms.

### Machine Learning and Statistical Analysis

We used a bootstrap aggregation (bagging) ensemble technique with reduced-error pruning regression tree as the underlying classifier to predict EE [12-15]. The bagging ensemble technique is presented here because it was superior to models generated using other techniques (eg, multilayer perceptron, support vector machines, linear regression, naïve Bayes, and reduced-error pruning regression trees). The bagging technique is an ensemble meta-algorithm to improve the stability and accuracy in statistical regression obtained by regression tree. The regression tree was built using information-theoretic criterion for selecting the nodes. Once the tree is built, reduced-error pruning is used, where each node, beginning with the leaves, is replaced with its most popular class. We divided the data for the model into n=10 folds, where, n−1 folds are for supervised learning and one fold is used to test the model for errors. The the value of errors obtained in a fold is added to the weights of the nodes of the next fold in the training set. A 10-fold cross validation was used to evaluate the model in order to ensure that the model was tested on data that it had not seen while training to minimize chance for overfitting. Data processing was done in MATLAB version 8.1.0.604 (R2013a) (MathWorks), and data mining (machine-learning algorithms) was done using Weka (Waikato Environment for Knowledge Analysis) software version 3.6.10.

### Existing Algorithms

**Figure 1:**
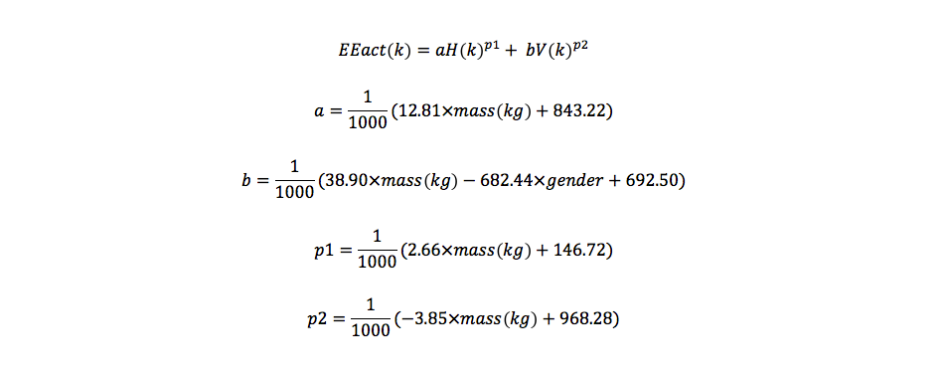
Resulting activity energy expenditure (EEact) using generalized nonlinear equation.

We used generalized nonlinear equations [16] originally developed based on the Tritrac-R3D accelerometer and verified with Actigraph, where H and V are the horizontal and vertical accelerometer-based counts, respectively, for the k-th minute and a, b, p1, and p2 are the generalized parameters that are modeled based on the subject’s gender (p1=male, p2=female) and mass in kg (Figure 1).

The resulting activity energy expenditure (EEact) is the amount of energy expended in kJ above resting energy expenditure (NOR-CHEN). For comparison with normal adults, we used a model developed from experiments on 23 healthy people. The model to estimate EE in healthy adults combined accelerometer and heart rate measurements; a protocol similar to the one outlined in this paper was followed for normal adults: obtaining sensor values and COSMED readings. In that analysis, two models were developed: one using linear regression (NOR-LIN) and the other using ensemble bagging technique over normal adults’ data (NOR-ENS). Further details of the healthy adult EE study are the subject of a different paper currently under review. Based on ambulatory data collected from young controls, we develop linear (regression) and nonlinear (machine-learning–based) models for EE estimation. YOU-LIN refers to the linear regression model developed based on young controls data and YOU-ENS refers to the model built on regression trees based on reduced-error pruning.

## Results

### Subject Characteristics

Physical characteristics of the subjects are shown in Table 1. All subjects completed the study protocol without any problems.

**Table 1:** Characteristics of subjects in the study.

Attributes	DMD boys n=7 mean (SD)	Child controls n=6 mean (SD)	Adult controls n=22 mean (SD)
Age, year	8.30 (1.70)	8.58 (1.35)	37.41 (13.61)
Height, cm	121.41 (10.43)	129.40 (0.09)	170.42 (8.51)
Weight, kg	28.72 (5.84)	26.25 (4.01)	73.52 (15.32)
BMI, kg/m^2^	19.32 (2.14)	15.69 (0.33)	25.14 (3.90)
Fitness: 6 min walk test, m	120.69 (16.34)	508.3 (57.5)	—

**Table 2:** Characteristics of the subsets of adult controls.

Characteristics	Youth mean	Middle age mean	Seniors mean
Age, years	23	34.51	54.94
Weight, kg	69	75.62	73.28
Height, cm	171.80	171.54	167.55

The adult controls were subsequently divided into three subgroups (see Table 2) to represent youth (aged 13-27 years), middle age (aged 28-50 years), and seniors (aged 50 years and older).

In our prior conference publication [17], we referred only to adult controls (n=22). The difference in population size between adults and boys with DMD could lead to potential bias, so we added control children of the same age group and divided the adult controls into three groups for comparison.

### Feature Selection

**Figure 2:**
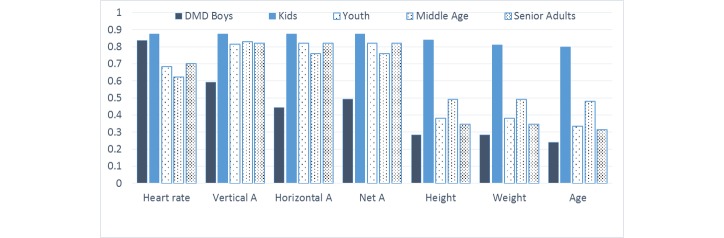
Relative information gain of different attributes on the energy estimation.

The goal of feature selection is to reduce the number of attributes used in the model and understand the predictive power of the original set of attributes. Correlation feature selection (CFS) was used to identify a subset of attributes for reduction of input attributes [18]. Age; height; weight; heart rate; and horizontal, vertical, and net acceleration measurements were retained, while BMI, recovery heart rate, and 6-minute–walk test values were removed. For the CFS technique used to determine subset of important features, see Multimedia Appendix 1. Figure 2 shows the plot of information gain (IG) for all of the attributes and leads to following observations:

For boys with DMD, heart rate readings have the highest IG contribution to EE estimation. Heart rate sensor outputs give higher IG regarding EE than measures such as age, weight, height, or accelerometer values.

The IG of heart rate measurements is similar for healthy children (controls) and children with DMD, but it is lower for elder controls in our study.

The accelerometer sensor has high correlation to EE in controls across all ages but low correlation for boys with DMD. This can be attributed to restricted ambulatory movement as well as inadequacy of a single accelerometer in capturing body acceleration of boys with DMD.

The demographic variables such as height, weight, and age have low correlation to EE in healthy adults and boys with DMD but high correlation for control children. This implies that knowing the demographics of healthy children—but not boys with DMD and adult controls—is helpful to EE estimation. We may need to investigate this further with a larger population of control children.

In the DMD group, accelerometer values (net A, horizontal A, and vertical A) have lower relative information contributions for determination of overall EE compared to normal adults where accelerometer readings have higher impact than heart rate. Other factors such as age, weight, and height have small IG for both populations. The reduced predictive power of smartphone accelerometer readings can be attributed to the unique body movement of DMD patients, making it impossible for a single accelerometer to capture their body motion effectively.

### Ensemble Model

Using the data obtained from the DMD children, we identified 11 attributes (10 input features and 1 output attribute) and 7560 total instances to develop a new model of EE. The 10 input features are as follows:

- Age
- Gender
- Weight
- Net acceleration (A) of accelerometer
- Net horizontal acceleration (H) of accelerometer
- Net vertical acceleration (V) of accelerometer
- Heart rate (HR)
- Product of HR and weight (HR×W)
- Product of net acceleration with weight (A×W)
- Product of net acceleration with height (A×H)

The attribute selection algorithm, based on CFS subset evaluation and best first search [13], was used to reduce input features and select the best features. Only 5 were selected and used in final algorithm: age, HR, HR×W, A×W, and A×H. We used the bagging ensemble technique with a reduced-error pruning regression tree as the underlying regression model to predict the EE values. The regression model generated from this choice outperformed others in terms of output correlation (91.21%) and mean absolute error (0.012): neural networks (84.63%, 0.020), linear regression (81.12%, 0.019), decision stump trees (58.01%, 0.025), stacking (0.03%, 0.030), and additive regression (78.73%, 0.022). This newly developed algorithm (DMD-ENS) builds a regression tree using information variance and prunes it using reduced-error pruning (with backfitting). DMD-NOR refers to the model built over DMD population but using simple linear regression instead of ensemble technique.

### Comparison With Existing Algorithms

**Figure 3:**
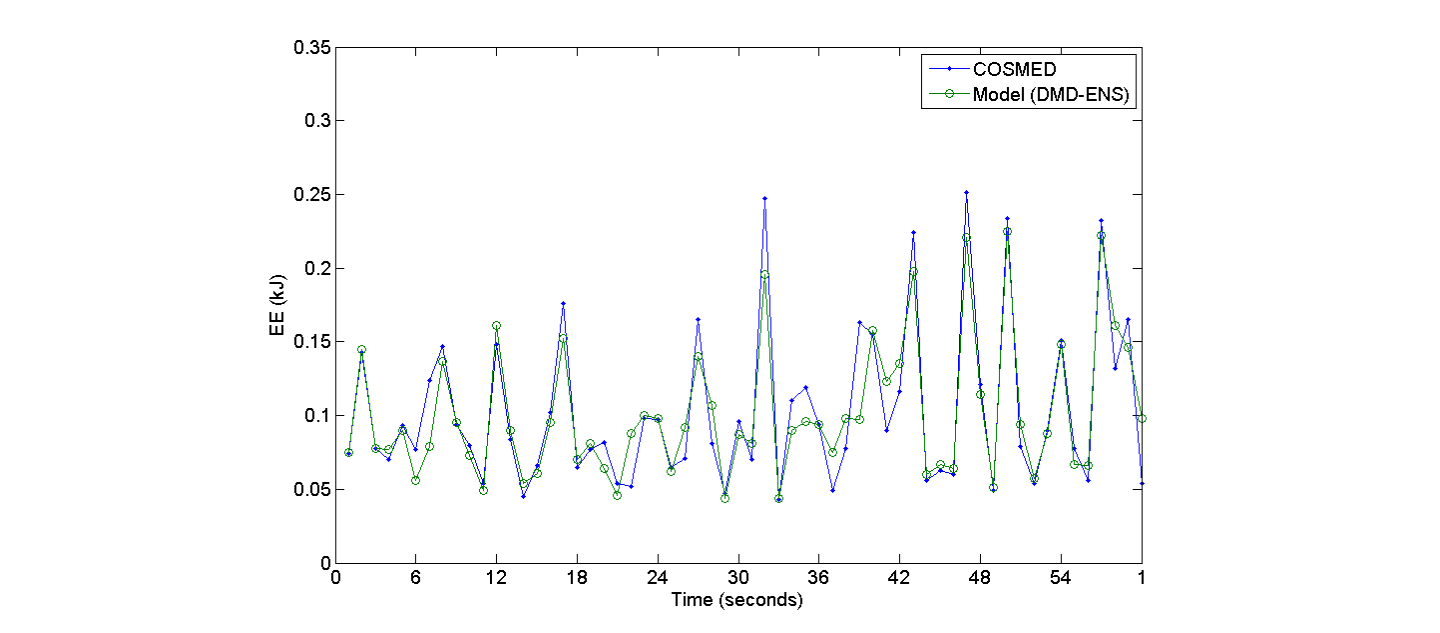
Plot showing energy estimation values obtained by COSMED and those estimated by ensemble model for DMD patients.

Results from the performance of the DMD-ENS and DMD-NOR models compared with models built over normal adults are shown in Table 3. It can be seen that existing adult models give a very poor performance (only 40% correlation) and a root mean square error (RMSE) of 0.05 to 0.75. Figure 3 gives a snapshot of EE values obtained from our ensemble model versus the actual reference values.

**Figure 4:**
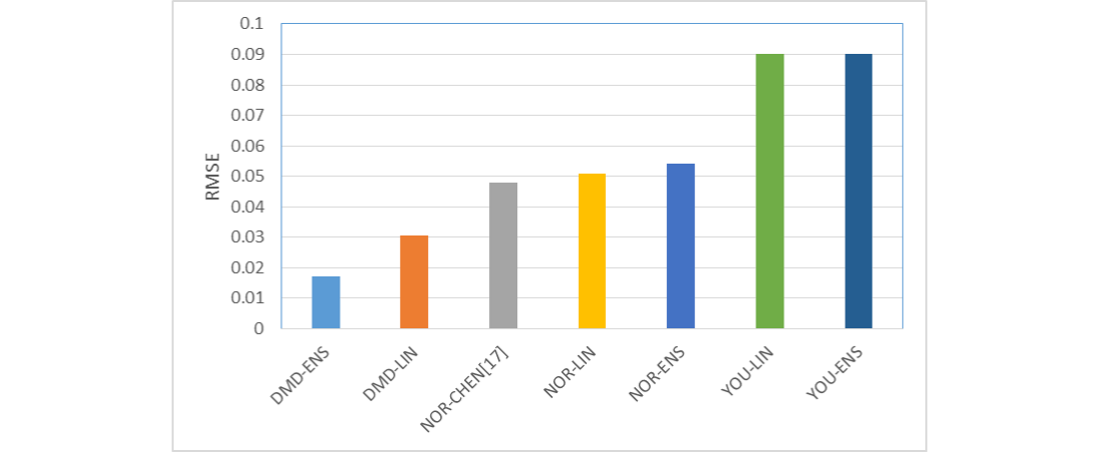
Bar chart showing root mean square error obtained using different models.

In our range of observations, the mean value of COSMED readings over the sample population (over 1 second epoch) was 0.09. Thus, an error of 0.03 is 33% and significant. The RMSE values are plotted in Figure 4.

**Table 3:** Performance comparison of DMD-ENS model with models for normal adults.

Model	Correlation to EE	Root Mean Square Error
DMD-ENS	91.20%	0.017
DMD-LIN	65.93%	0.031
NOR-CHEN [16]	40.62%	0.048
NOR-LIN	41.59%	0.051
NOR-ENS	37.91%	0.054
YOU-LIN	31.22%	0.723
YOU-ENS	46.75%	0.182

## Discussion

### Principle Findings

We found that existing models gave poor correlation (40%) and high error in estimating EE for children with disability. Next, we explored the role of innovative machine learning with data collected from these sensors to obtain an accurate EE model. The nonlinear machine-learning–based approach to estimate EE for children with DMD uses reduced-error pruning for regression trees with ensemble bagging models and gives high correlation (91.21%) and an RMSE of 0.017.

In this work, we explored using machine-learning techniques over data from accelerometer and heart rate sensors to obtain an accurate EE model for children with disabilities. Compared to the EE data obtained from the COSMED K4b2, EE estimation based on our proposed model (DMD-ENS) has high correlation and can be obtained by simple body-worn accelerometer and heart rate sensors, which are becoming more and more popular with new emerging wearable devices such as Fitbit, Apple Watch, and Microsoft Band. Although these devices use proprietary algorithms, the algorithms are based on machine-learning models built for different activities of daily living [19]. In our prior work, we have shown that the machine-learning models developed in the lab can outperform these algorithms for specific ambulatory movements [20]. The poor performance of algorithms for the healthy population (only 40% correlation) indicates that these devices are not ready to use for measuring physical activity in populations with muscular dystrophy. The high correlation of a custom machine-learning model built over a dataset from children with disabilities, however, shows feasibility of developing population-specific models for EE estimation. In our future work, we would like to conduct trials over a large sample size with a larger set of ambulatory activities.

While this single model appears to work across a range of activities in a clinical setting, further investigation into the validity of this EE estimation model for daily activities outside of the clinic is needed. We observed that the existing models, developed based on adult populations, do not provide accurate levels of EE estimates. When we built regression models on healthy children (controls), we realized that these models do not extend to children with disabilities. It is not merely the age of subjects but also their gait and other aberrations which affect EE for populations with muscular dystrophy. This confirms our assertion that population-specific models are required for EE estimation and a generic framework will not work. We also need to expand our population base to include children with other forms of muscular dystrophy to see if our proposed model scales well to those populations.

Further investigation into the bodily placement of multiple sensors will add to the information gained by sensors in specific bodily locations. Boys with DMD perform a high number of compensatory movements to walk and cover shorter distances; it would be possible to infer that using multiple accelerometers would detect such movements and this could be a confounding factor. In this study, we placed a single accelerometer sensor at the waist of the boys with DMD and found that waist acceleration is not a good predictor for EE. It is conceivable that information from multiple sensors will increase accuracy of this EE model for disabled populations depending on the particular conditions of the disability and impairment. Sensors placed on multiple body locations may be able to capture all dimensions of body motion and energy expenditure. Recent work [8] uses videotape analysis of DMD patients to develop a functional evaluation scale of gait for DMD. Sensor-based models can be used to augment functional evaluation scales in understanding progression of the disease.

Most of the participants found the sensors easy to use and unobtrusive and would be willing to wear them on a daily basis as a tool to monitor physical activity and energy balance as part of their treatment program.

### Limitations

Sample size was small due to the limited size of the DMD population accessible and willing to participate in our study. We plan to continue collecting data from DMD patients to validate our results. A second limitation is that laboratory-based measurements may not correlate to regular daily activity and should be further validated in home or community settings.

### Conclusion

The experiments show that machine-learning models developed for healthy populations are inaccurate for children with disabilities. An ensemble machine learning technique (bagging) based on combined accelerometer and heart rate sensor readings gave high accuracy (91.21%) to actual EE. The results are encouraging and will be useful to track energy expenditure of large patient populations in field activities.

## Multimedia Appendix 1

Correlation feature selection.

## Abbreviations

A×W: product of net acceleration and weight
A×H: product of net acceleration and height
CFS: correlation feature selection
DMD: Duchenne muscular dystrophy
EE: energy estimation
HR×W: product of weight and heart rate
IG: information gain
RER: respiratory exchange rate
REE: resting energy expenditure

## References

[1] Wu W, Dasgupta S, Ramirez EE, Peterson C, Norman GJ (2012). Classification accuracies of physical activities using smartphone motion sensors. J Med Internet Res.

[2] Fanning J, Mullen S, McAuley E (2012). Increasing physical activity with mobile devices: a meta-analysis. J Med Internet Res.

[3] Kirwan M, Duncan M, Vandelanotte C, Mummery W (2012). Using smartphone technology to monitor physical activity in the 10,000 Steps program: a matched case-control trial. J Med Internet Res.

[4] McDonald CM (2002). Physical activity, health impairments, and disability in neuromuscular disease. Am J Phys Med Rehabil.

[5] Westerterp KR (2009). Assessment of physical activity: a critical appraisal. Eur J Appl Physiol.

[6] Crouter SE, Churilla JR, Bassett DR (2008). Accuracy of the Actiheart for the assessment of energy expenditure in adults. Eur J Clin Nutr.

[7] Eagle M, Baudouin SV, Chandler C, Giddings DR, Bullock R, Bushby K (2002). Survival in Duchenne muscular dystrophy: improvements in life expectancy since 1967 and the impact of home nocturnal ventilation. Neuromuscul Disord.

[8] Duffield R, Dawson B, Pinnington HC, Wong P (2004). Accuracy and reliability of a Cosmed K4b2 portable gas analysis system. J Sci Med.

[9] Fujiwara SM, Komaki H, Nakagawa E, Yoshimura M, Oya Y, Fujisaki T, Tokita Y (2012). Decreased resting energy expenditure in patients with Duchenne muscular dystrophy. Brain Dev-JPN.

[10] Elliott SA, Davidson ZE, Davies PSW, Truby H (2012). Predicting resting energy expenditure in boys with Duchenne muscular dystrophy. Eur J Pediatr Neurol.

[11] Souza M, Ferreira ME, Baptista A, Sverzut ACM (2014). Gait energy expenditure in children with Duchenne muscular dystrophy: case study. Fisioterapia Pesquisa.

[12] Breiman L (1996). Bagging predictors. Machine Learning.

[13] Witten I, Frank E, Hall MV (2005). Data Mining: Practical Machine Learning Tools and Techniques, Third Edition.

[14] Gopalakrishnan K, Agrawal A, Ceylan H, Kim S, Choudhary A (2013). Knowledge discovery and data mining in pavement inverse analysis. Transport.

[15] Mathias J, Agrawal A, Feinglass J, Cooper AJ, Baker DW, Choudhary A (2013). Development of a 5 year life expectancy index in older adults using predictive mining of electronic health record data. J Am Med Inform Assn.

[16] Donairs-Gonzalez D, de Nazelle A, Seto E, Mendez M, Nieuwenhuijsen M, Jerrett M (2013). Comparison of physical activity measures using mobile phone-based CalFit and Actigraph. J Med Internet Res.

[17] Pande A, Casazza G, Nicorici A, Seto E, Miyamoto S, Lange M, Abresch R, Mohapatra P, Han J (2014). Energy expenditure estimation in boys with Duchenne muscular dystrophy using accelerometer and heart rate sensors.

[18] Hall MA (1999). Correlation-based feature selection for machine learning [dissertation].

[19] Dannecker K, Petro SA, Melanson EL, Browning RC (2011). Accuracy of fitbit activity monitor to predict energy expenditure with and without classification of activities. Med Sci Sport Exer.

[20] Pande A, Zeng Y, Das A, Mohapatra P, Miyamoto S, Seto E, Henricson E, Han J (2013). Energy expenditure estimation with smartphone body sensors.

