# Supplementary material for: Machine Learning to Improve Energy Expenditure Estimation in Children With Disabilities: A Pilot Study in Duchenne Muscular Dystrophy
Source: JMIR Rehabil Assist Technol. 2016 Jul 19;3(2):e7. doi: 10.2196/rehab.4340 (PMC5454548; doi:10.2196/rehab.4340)
Supplement: Supplementary file 1 [file rehab_v3i2e7_app1.pdf]

Multimedia Appendix 1

The CFS technique was used with a greedy stepwise search to find the subset S with the best average merit, which is given by Ms.

$$M_s = \frac{n r'_{P_D}}{\sqrt{n + n(n-1) r'_{P_f}}}$$

Where n is the number of features in S, r'_{PD} is the average value of feature-outcome correlations, and r'_{Pf} is the average value of all feature-feature correlations. We used the information gain metric to measure the relative predictive power of each final attribute in our dataset, training set “T” [14]:

$$IG(T, A) = H(T) - H(T \vee A)$$

H() represents the information entropy and is an attribute.
